# Reverse-Phase Protein Microarrays for Overexpressed *Escherichia coli* Lysates Reveal a Novel Tyrosine Kinase

**DOI:** 10.1021/acs.analchem.4c00965

**Published:** 2024-04-29

**Authors:** Batuhan Birol Keskin, Chien-Sheng Chen, Pei-Shan Tsai, Pin-Xian Du, John Harvey M. Santos, Guan-Da Syu

**Affiliations:** † Department of Biotechnology and Bioindustry Sciences, National Cheng Kung University, Tainan 701, Taiwan; ‡ Department of Food Safety/Hygiene and Risk Management, College of Medicine, National Cheng Kung University, Tainan 701, Taiwan; § Institute of Basic Medical Sciences, College of Medicine, National Cheng Kung University, Tainan 701, Taiwan; ∥ Centre for Animal Science, Queensland Alliance for Agriculture and Food Innovation, The University of Queensland, Brisbane, QLD 4072, Australia; ⊥ International Center for Wound Repair and Regeneration, National Cheng Kung University, Tainan 701, Taiwan; # Medical Device Innovation Center, National Cheng Kung University, Tainan 701, Taiwan

## Abstract

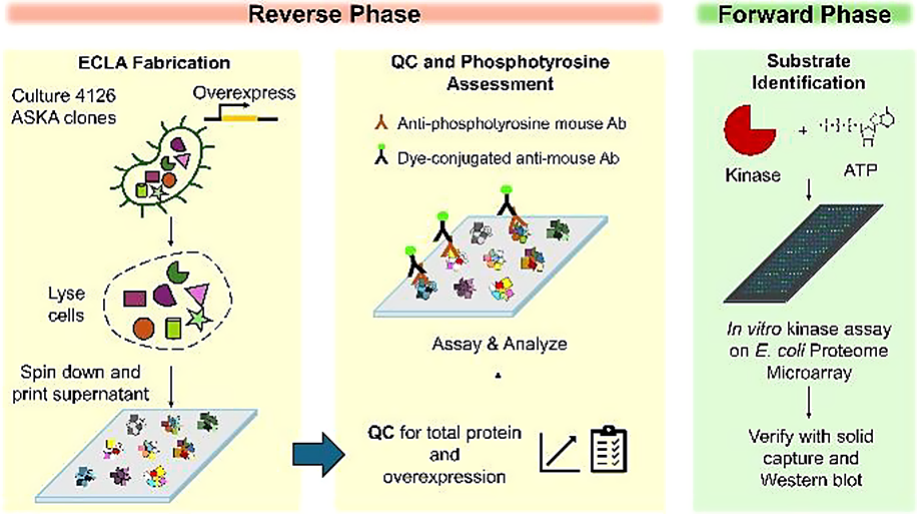

Tyrosine phosphorylation is one of the most important posttranslational modifications in bacteria, linked to regulating growth, migration, virulence, secondary metabolites, biofilm formation, and capsule production. Only two tyrosine kinases (yccC (etk) and wzc) have been identified in *Escherichia coli*. The investigation by similarity has not revealed any novel BY-kinases *in silico* so far, most probably due to their sequence and structural variability. Here we developed a reverse-phase protein array from 4126 overexpressed *E. coli* clones, lysed, and printed on coated glass slides. These high-density *E. coli* lysate arrays (ECLAs) were quality controlled by the reproducibility and immobilization of total lysate proteins and specific overexpressed proteins. ECLAs were used to interrogate the relationship between protein overexpression and tyrosine phosphorylation in the total lysate. We identified 6 protein candidates, including etk and wzc, with elevated phosphotyrosine signals in the total lysates. Among them, we identified a novel kinase nrdD with autophosphorylation and transphosphorylation activities in the lysates. Moreover, the overexpression of nrdD induced biofilm formation. Since nrdD is a novel kinase, we used *E. coli* proteome microarrays (purified 4,126 *E. coli* proteins) to perform an in vitro kinase assay and identified 33 potential substrates. Together, this study established a new ECLA platform for interrogating posttranslational modifications and identified a novel kinase that is important in biofilm formation, which will shed some light on bacteria biochemistry and new ways to impede drug resistance.

## Introduction

Protein phosphorylation is one of the most studied posttranslational modifications (PTMs) in bacteria as they have crucial regulatory functions. Protein kinases are essential effectors of protein phosphorylation. Bacterial tyrosine kinases, also known as BY-kinases, are generally involved in the biosynthesis, polymerization, and export of complex polysaccharides necessary for the creation of biofilm or capsules.^1^ Until now, yccC (etk) and wzc are the only two identified BY-kinases in *Escherichia coli* (*E. coli*).^2,3^ The presence of unknown BY-kinase in the *E. coli* proteome has been suggested, as the Δ*etk*-Δ*wzc* double mutants still have tyrosine-phosphorylated substrates. Nevertheless, structure-based *in silico* search has not revealed any additional BY-kinases in *E. coli*.^4^

Identification of kinases in prokaryotes is challenging as they have less specific similarities to their eukaryotic counterparts and are sometimes hidden in the genome.^5^ The motifs already identified for protein kinases might be too stringent, as some of the discovered protein kinases lack conserved motifs.^6^ High-throughput screening of differentially phosphorylated proteins over the whole proteome is a viable approach to interrogate kinases. Nowadays, phosphoproteomics analysis via mass spectrometry (MS) is a tool to reveal the in vivo substrates and characterization of a kinase. However, MS is laborious and has low sample throughput in parallel.^7,8^ Moreover, there are no existing high-throughput tools to identify or screen for novel kinases. Therefore, to be able to perform proteome-wide kinase screening, practical and economical approaches are needed.

Functional screening of each gene can be accomplished by overexpressing each gene. Kitagawa et al. established the ASKA library as a collection of open reading frames cloned into *E. coli* K-12 and can be used to overexpress His-tagged proteins.^9^ By purifying every His-tagged protein, Chen et al. established the *E. coli* proteome microarray as a high-throughput screening platform. Since kinase needs substrates to perform phosphorylation,^10,11^ this type of purified *E. coli* proteome microarray (forward-phase) cannot be used to identify kinases. Reverse-phase protein array (RPPA) is a microarray that immobilizes hundreds or thousands of unpurified protein mixtures. The RPPA can be used to identify a specific protein expression in all the lysates that are printed on the array.^12^ The RPPA has been used to profile the histone modifications in the nuclear extracts or cell lysates.^13,14^ Since the cell lysates on the RPPA array can serve as the substrates, we propose that the overexpression of a kinase may increase the phosphorylation of the substrates and thus can be screened for kinases. In our study, we constructed RPPA with lysates of 4126 different overexpressed strains from the ASKA library and named *E. coli* lysate arrays (ECLAs). We profiled phosphotyrosine levels in ECLAs by antiphosphotyrosine antibodies and obtained 6 BY-kinase candidates. We further identified nrdD as a novel BY-kinase and used the purified *E. coli* proteome microarray (forward-phase) to report the specific substrates.

## Materials and Methods

### Strains and Bacterial Culture

ASKA *E. coli* open reading frame (ORF) library strains^9^ and wild-type (WT) *E. coli* K-12 strain BW25113 were used in the study.^15^ 4126 ASKA strains were inoculated from 56 plates that were available to us from the ASKA library and cultured overnight in Luria–Bertani (LB) medium with 30 μg/mL chloramphenicol and grown in an incubator at 37 °C, 250 rpm in 96-deep well plates. All cultures were brought to OD_600_ 0.1, induced by 1 mM isopropyl-*b*-D-thiogalactopyranoside (IPTG) (no. I6758, Sigma-Aldrich), and cultured for another 4 h to induce protein expression.

### Lysate Preparation and ECLA Fabrication

Cells in 96-deep well plates were harvested by centrifugation at 4121×*g* for 10 min and frozen immediately at −80 °C. Harvested pellets were resuspended in B-PER lysis buffer (no. 78248, Thermo Scientific), 0.5 mg/mL lysozyme (no. 62971, Sigma-Aldrich) phosphatase inhibitor (1 tablet per 10 mL, no. A32957, Thermofisher) and protease inhibitor cocktail (1 tablet per 10 mL, no. #11836153001, Roche). After vortexing for 2 min and incubating for 8 min at room temperature (RT), the mixtures were sent to a centrifuge at 4121×*g* for 10 min. Lysates were transferred to 384-well plates, supplemented with glycerol, and stored at −80 °C for printing. Each lysate was spotted in duplicates on aldehyde-coated slides using a SmartArrayer 48 microarrayer (CapitalBio).^16^ The printed microarrays were stored at −80 °C for further usage.

### Quality Control of ECLA

The quality of ECLA was evaluated by the amount of lysate immobilized, the reproducibility between different batches, and the amount of protein immobilized. To monitor the immobilization of the lysates on the ECLAs, arrays were incubated with 1.66 μg/mL DyLight 650 NHS Ester (no. 62265, Thermo Fisher Scientific) in phosphate-buffered saline with 0.1% Tween 20 (PBST) and shaken for 1 h at 50 rpm. After washing 3 times with PBST, slides were spin-dried and scanned by a laser scanner (#Spinscan HC-BS01, CADUCEUS Biotechnology). The fluorescence intensities were equivalent to the protein amounts which can be used to visualize the immobilization status of lysates on arrays.^17^ To determine the protein overexpression in the lysates, ECLAs were blocked with 3% bovine serum albumin (BSA) in Tris-buffered saline with 0.1% Tween 20 (TBST) and incubated with 1 μg/mL of Cy3-labeled anti-6xHis antibody (#200-304-382, Rockland) in 1% BSA in TBST for 1 h with 50 rpm shaking. After 3 washes, the slides were dried and scanned.

### Tyrosine Phosphorylation Profiling on ECLA

ECLAs were blocked for 1 h with 50 rpm shaking and incubated with 100x diluted phosphotyrosine mouse mAb (P-Tyr-100, #9411, Cell Signaling) in 1% BSA in TBST for another hour. Arrays were washed three times and then incubated with fluorescence-labeled goat antimouse secondary antibody (0.25 μg/mL, #84540, Thermo Scientific) for 1 h with shaking. Another antiphosphotyrosine antibody (100× dilution, #ab10321, Abcam) was used with similar assay procedures except the concentration of the secondary antibody changed to 0.5 μg/mL. After three final washes, the arrays were dried and scanned. Control experiments were performed using the same procedures without adding the primary antibodies. The phosphotyrosine signals were calculated based on foreground minus background. The three selection criteria for the outstanding phosphotyrosine spots were *p* < 0.05 between experiment and control groups, 1.5 standard deviations (SDs) differences compared to WT lysates and overlapped with two antiphosphotyrosine antibodies.

### *In Vitro* Kinase Assays

His_6_-tagged proteins were IPTG-induced, lysed, and purified with Ni-NTA resin as described earlier.^18^ The purified protein concentration was calculated using BSA standards on SDS-PAGE. Autophosphorylation assay is carried out in kinase buffer (no. 9802, Cell Signaling) with 0.4 mM ATP (no. 9804, Cell Signaling) with purified proteins at 30 °C for several time points. The reaction was terminated by adding SDS loading buffer (5× dilution, 10% SDS, 50% Glycerol, 0.05 M DTT, 0.01 EDTA, 0.05% bromophenol blue, and 0.125 M Tris–HCl, pH: 6.8). The substrate phosphorylation was performed with 4 μg of ssuD and 4 μg nrdD. A total reaction volume of 20 μL was prepared with and without 0.4 mM ATP, and with and without nrdD in kinase buffer at 30 °C for 1 h. The reaction was terminated by adding an SDS loading buffer. *In vitro* kinase assays were also performed by adding purified kinases into WT lysates with 0.4 mM ATP in kinase buffer at 30 °C for 1 h.

For the solid capture experiment, 1 μg of ssuD and 100 μL of carbonate/bicarbonate buffer (pH: 9.6) were adsorbed to each 96-well overnight at 4 °C with 200 rpm shaking. The wells were washed with TBST 4 times. The kinase reaction was performed by adding 8 μg of nrdD with and without 0.4 mM ATP in kinase buffer at 30 °C for 1 h. The wells were washed by TBST 4 times and blocked for 1 h in RT by shaking at 50 rpm for 1 h in 3% BSA in TBST. Wells were incubated with 200 μL of phosphotyrosine mouse mAb (500× dilution, P-Tyr-100) with 1% BSA in TBST. After incubating for 1 h at room temperature with shaking, wells were washed with TBST and then incubated with 200 μL of peroxidase-conjugated goat anti-mouse IgG (2000× dilution, #115-035-003, Jackson Immunoresearch). After the final washes, TMB substrates (#421101, BioLegend) were added followed by a stop solution (2 N H_2_SO_4_). The absorbance was recorded at the OD_450_ by a microplate reader.

### Western Blot Assays

Protein samples or lysate mixtures were run on 4–12% gradient gels (#M00654, GenScript) and then transferred to a PVDF membrane at 200 mA for 1 h. Ponceau S staining was performed sometimes. For Pro-Q Diamond staining (#P33356, Thermo Fisher Scientific), membranes were stained, washed, and imaged according to the manufacturer’s protocol. For total protein Coomassie brilliant blue (CBB) staining was performed for 2 min, destained with the destaining solution for 5 min 2 times, and imaged. Phos-tag staining (#BTL-111S1, NARD Institute) was performed based on the manufacturer’s protocol.^19^ Briefly, membranes were blocked, washed, incubated with Phos-tag, washed, incubated with streptavidin-conjugated horseradish peroxidase streptavidin, washed, incubated with substrates, and then imaged. For antibody detections, membranes were blocked in 3% BSA for 1 h, washed, incubated with phosphotyrosine mouse mAb (3000×, P-Tyr-100) in 1% BSA for 1 h, washed, incubated with peroxidase-conjugated goat anti-mouse IgG (6000× dilution) for 1 h, washed, incubated with substrates (#WBKL50500, Merck), and imaged by Chemidoc system (#12003153, BIO-RAD). Similar procedures were used in other antibodies, except the concentration of primary antibodies, e.g., phosphoserine/threonine antibody (3000× dilution, #PM3801, ECM Biosciences) and antiphosphohistidine antibody (3000× dilution, #MABS1351, Sigma-Aldrich).

### Phosphorylation Profiling on *E. coli* Proteome Microarrays

*E. coli* proteome microarrays were prepared and fabricated as previously described.^20^ The arrays were blocked in 3% BSA in TBST for 1 h at RT. The kinase reactions were prepared by adding 20 μg nrdD in 1% BSA kinase buffer with or without 0.2 mM ATP. Reactions were then applied to each array in duplicates under the coverslips and incubated in a humidified chamber at 30 °C for 1 h. The arrays were washed twice, probed with 250 μL phosphotyrosine mouse mAb (1000× dilution) in 1% BSA in TBST for 1 h, washed three times, and then probed with 0.125 μg/mL of fluorescence-labeled goat antimouse antibody for 1 h. After three final washes, arrays were dried and scanned. The substrate selection criteria for the outstanding phosphotyrosine spots were *p* < 0.05 and 1 SD difference between with and without ATP.

### Biofilm Formation Assay

Overnight grown nrdD strains in 30 μg/mL chloramphenicol-supplemented LB were brought to 0.1 OD_600_ and grown until OD_600_ 0.6. The nrdD strains were transferred into a 96-well microplate with 100× dilution in 200 μL LB in five replicates with and without 0.2 mM IPTG. The biofilm assay was performed by adapting the microplate method described previously.^21^ After growth for 18 h at 37 °C, OD_600_ was recorded to normalize the cell counts. Plates were washed twice with RO water, and 300 μL of 0.1% crystal violet was applied for 20 min in RT to stain the remaining *E. coli*. After 2 washes, 300 μL 95% ethanol was added to the wells and incubated for 20 min in RT. The OD was measured at 540 nm absorbance. The biofilm formation is calculated as OD_540_/OD_600_ value.

### Data Analysis and Bioinformatics

Foreground and background fluorescence was obtained after spot alignment with GenePix Pro 6.0 software (Molecular Devices). Fluorescence intensities were calculated based on the foreground minus the background. The student’s *t*-test was used to calculate the *p*-value between groups. *P* < 0.05 was defined as significant. All data were presented as mean ± SD nrdD homologous sequences were obtained with a protein BLAST search from the UniProt database. The sequences were aligned by the M-Coffee tool from the T-Coffee alignment server.^22^ Maximum likelihood analysis was performed with IQ-Tree web server.^23^ The phylogenetic tree was visualized by iTOL online tool.^24^ Percent identity and E values were obtained from NCBI BLAST protein alignments.

## Results

### Fabrication of ECLA and Quality Control

**Figure 1:**
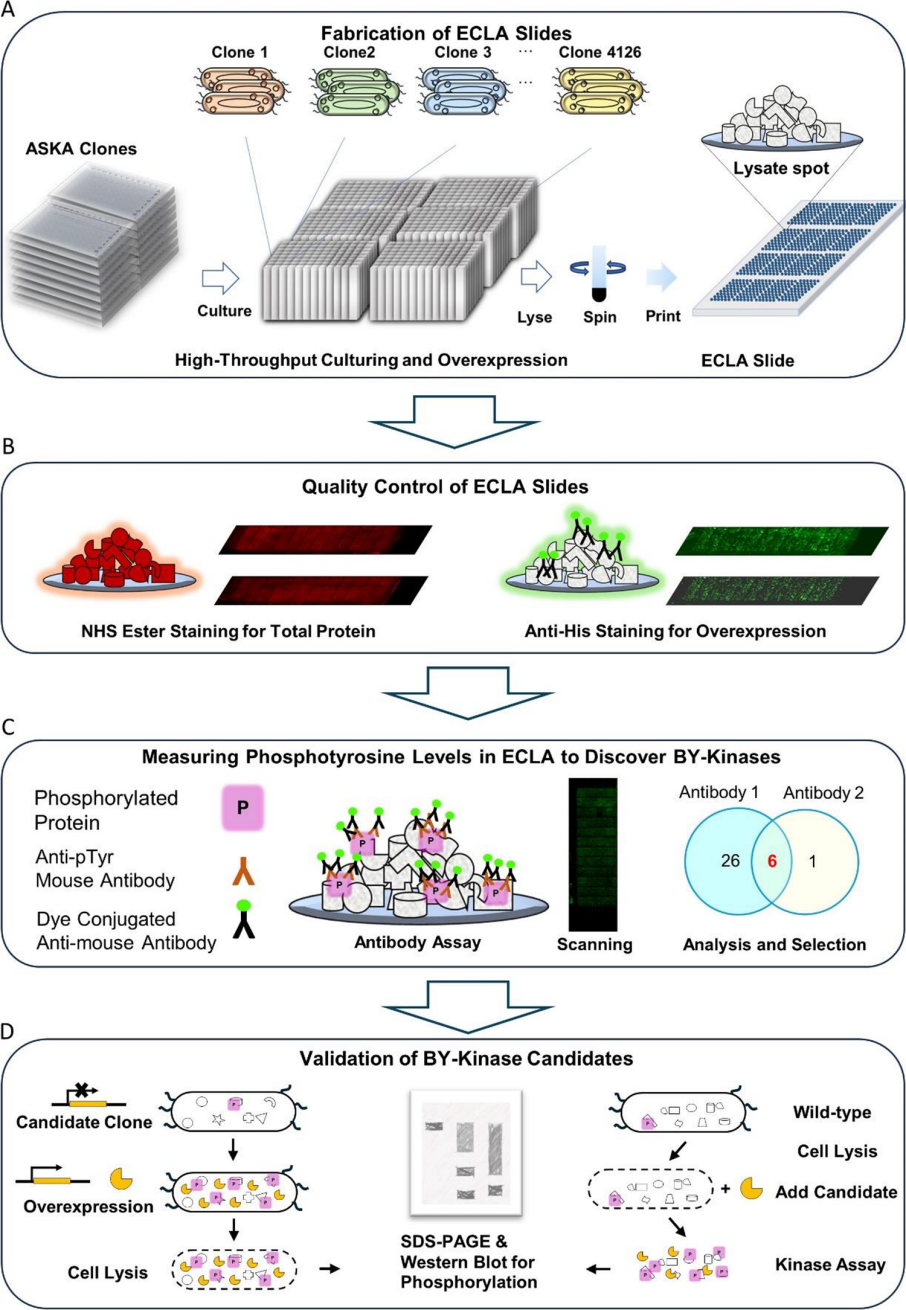
Workflow of BY-Kinase interrogation by the ECLA. The overall workflow of tyrosine phosphorylation profiling of ECLA starts with (A) ECLA fabrication by high-throughput culturing, induction, lysis, clarify, and printing of the 4126 overexpressed ASKA clones onto aldehyde-coated slides. (B) NHS succinyl ester Dylight 650 was used to visualize total protein on ECLA slides (left). Overexpression of 6xHis-tagged protein in the lysate spots was monitored by anti-His fluorescence staining (right). (C) Phosphorylation levels in each lysate clone were monitored by anti-pTyr antibody staining on ECLA. The BY-kinase candidates were selected by overlapping two different anti-pTyr antibodies. (D) BY-kinase candidates were verified by *in vivo* overexpressing (left) and *in vitro* addition (right). The level of phosphorylation in the lysates was monitored by Western blot.

To develop the ECLAs, we cultured 4126 *E. coli* ORF clones and induced them with IPTG to overexpress proteins. Cell cultures were collected by centrifugation and lysed by lysis buffer with phosphatase and protease inhibitors. After lysis, the clarified supernatants were harvested by centrifuge. All the culturing, induction, lysis, and clarification steps were carried out in the 96-well format. The clarified lysates were transferred into 384-well plates and printed onto aldehyde-coated slides in duplicate (Figure 1A). As the spots were prone to merge due to detergent in the lysis buffer, we optimized the spot distance and adjusted glycerol concentration to prevent merging during the printing.

**Figure 2:**
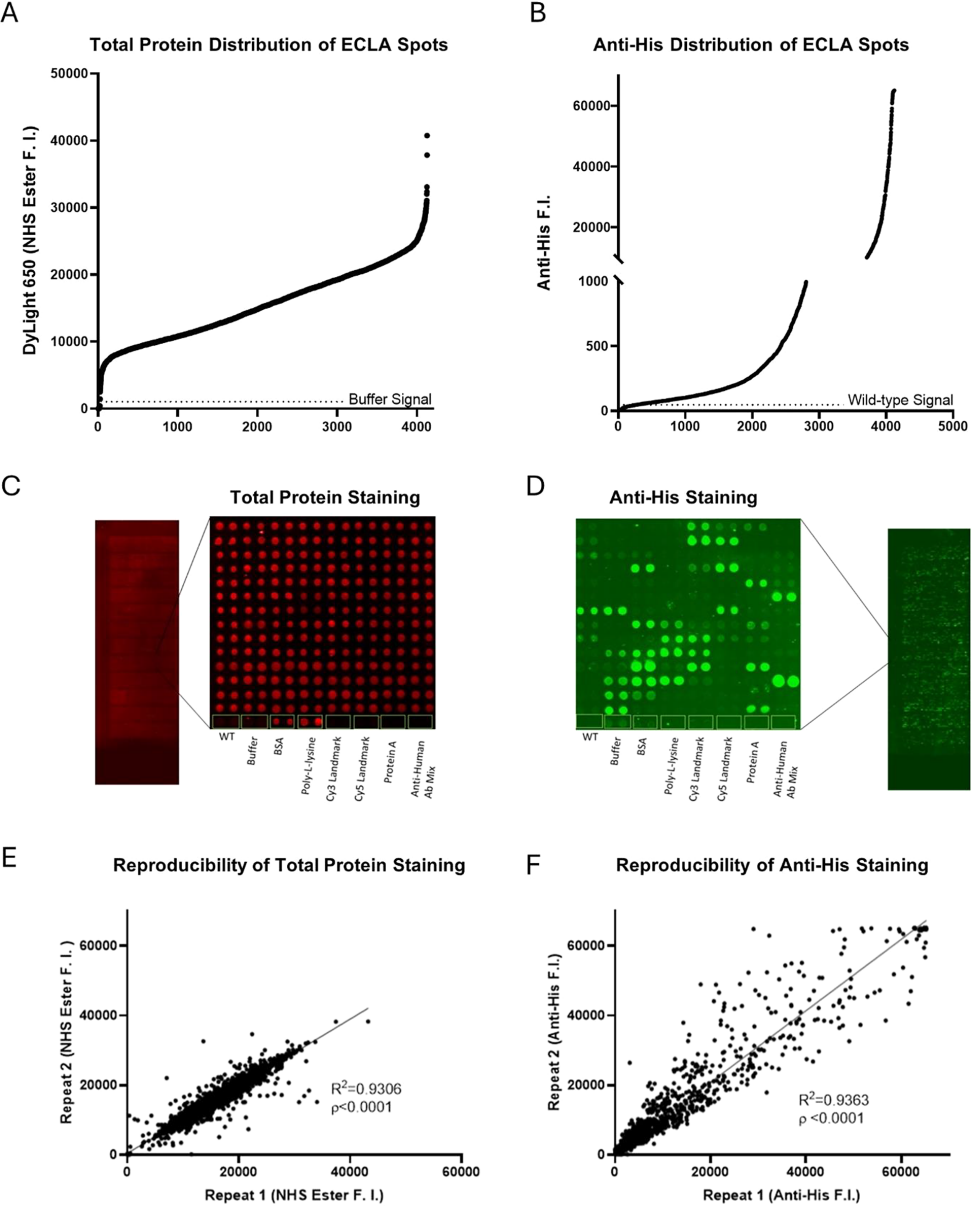
Quality control of ECLAs. Quality control of ECLAs was assessed by Dylight 650 NHS Ester for total protein (A,C,E) and anti-His for overexpressed protein (B,D,F). (A) Distribution of total protein from ECLA spots was obtained from two independent assays. Fluorescence intensity from the buffer was indicated with the dotted line. (B) Distribution of 6xHis-tagged protein from ECLA spots was obtained from two independent assays. Fluorescence intensities from WT lysate signals were indicated with the dotted line. (C) Representative image of an ECLA stained by Dylight 650 NHS Ester. (D) Representative image of an ECLA block stained by anti-His. Control spots were indicated in the bottom row. (E) Reproducibility of total protein staining obtained from two independent ECLA assays. (F) Reproducibility of overexpressed protein in the lysates obtained from two independent ECLA assays.

The quality control of the ECLAs was done by total protein staining with amine-reactive NHS ester and overexpressed protein staining with anti-His antibody in duplicate (Figure 1B). The fluorescence intensity for buffer spots was 995.29 in total protein staining. On the ECLAs, 99.39% of lysate spots have more fluorescence intensities than buffer spots in total protein staining (Figure 2A). As IPTG induced overexpression of His-tagged proteins, the overexpression level of lysate spots can be monitored by anti-His staining. As WT lysates did not contain His-tagged proteins, they can be used as the background with 45.82 fluorescence intensity in anti-His staining. On the ECLAs, 93.80% of lysate spots have His-tagged proteins compared to WT lysate spots in anti-His staining (Figure 2B). The control spots were used for alignment, and positive controls or negative controls were printed at the bottom of each block, containing WT lysate, lysis buffer, BSA, poly-l-lysine, Protein A, antihuman antibody, and fluorescence landmark (Figure 2C, D). The reproducibility of ECLAs was *R*^2^ = 0.9306 for total protein staining (Figure 2E) and *R*^2^ = 0.9363 for anti-His staining (Figure 2F).

### Profiling the Level of Tyrosine Phosphorylation in Cell Lysates by Using ECLAs

**Figure 3:**
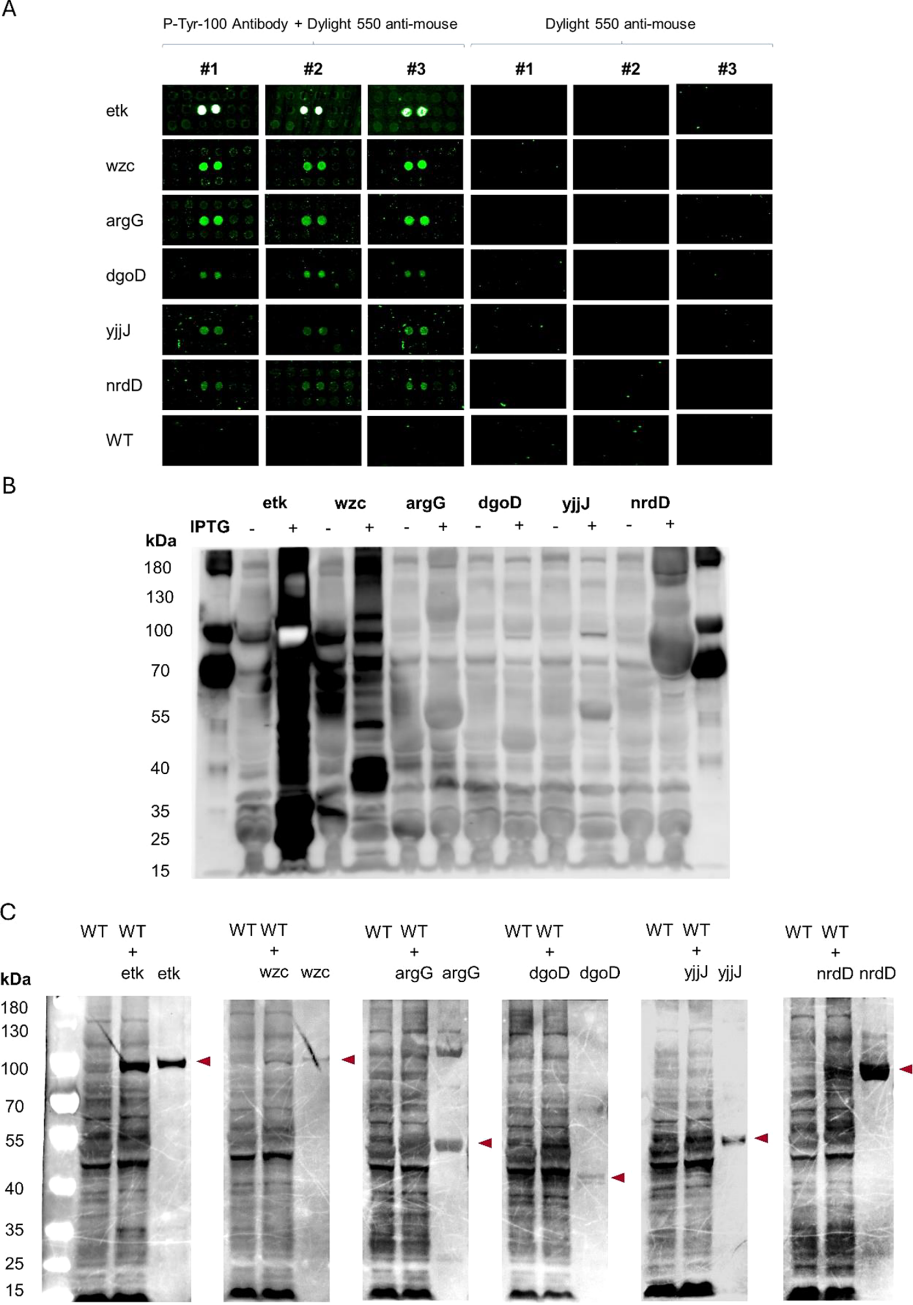
Lysate profiles of overexpressed candidates. There were 6 overlapped hits from the two anti-pTyr ECLA assays. (A) Images from the three assays were done with P-Tyr-100 antibody followed by fluorescent-labeled antimouse, and three assays were done with fluorescent-labeled antimouse as blanks. The WT lysate spots were listed as baseline phosphorylation. (B) Each ASKA clone was listed on top, including etk, wzc, argG, dogD, yjjJ, and nrdD. Immunoblots were performed with P-Tyr-100 antibody to show the phosphorylation profiles in the lysates with or without IPTG inductions. (C) Each purified candidate was incubated with or without WT lysates in the kinase buffer supplemented with ATP before analysis by SDS-PAGE. Pro-Q Diamond staining was used to image the phosphorylated levels.

Since the ECLAs immobilized thousands of lysates from overexpressing clones, they are ideal for profiling the phosphorylation networks because they contain both enzymes and substrates. To quantify the tyrosine phosphorylation on ECLAs, we selected two antiphosphotyrosine antibodies, P-Tyr-100 and PY20, to minimize the potential bias from the antibodies. The phosphotyrosine levels were then visualized with Dylight-conjugated antimouse antibodies. After scanning, alignment, and biostatistics, there were 32 significant hits from P-Tyr-100 (Table S1) and 7 significant hits from PY20 (Table S2). The overlapping between two phosphotyrosine antibodies was 6 hits, e.g., etk, wzc, argG, dgoD, yjjJ, and nrdD. The rank of each candidate hit for both antibodies can be found in Table S3. The representative images from the P-Tyr-100 assays (Figure 3A) and PY20 assays (Figure S1) including primary and secondary antibodies or secondary antibodies only were listed. These 6 hits were potential candidates for the BY-kinases because the overexpression resulted in the elevation of phosphotyrosine levels.

### Profiling Cell Lysates of BY-Kinase Candidates by Immuno-Western Blot

To further elucidate the BY-kinase activities in 6 candidates, we performed Western blots of cell lysates to show the phosphorylation patterns. Lysates from the overexpressed candidates were used to run SDS-PAGE followed by P-Tyr-100 antibody blot (Figure 1D, left). Each clone that contained candidate ORF was included for candidate protein expression with or without IPTG and further analyzed by a P-Tyr-100 antibody blot (Figure 3B). All IPTG-included clone lysates showed distinctive phosphorylated bands at different degrees compared to their non-IPTG controls. Overexpression of etk exhibited highly saturated signals across the whole lane, and overexpression of wzc had a few saturated bands (Figure 3B).

We analyzed the kinase activities by treating the purified candidates with WT lysates as substrates for *in vitro* kinase assays. To observe the phosphorylation patterns, we performed the kinase assay with ATP and visualized it with Pro-Q Diamond staining for the WT lysate, WT lysate treated with purified candidates, and purified candidates (Figure 3C). Based on the blot images, etk, wzc, yjjJ, and nrdD showed distinct band formations or increased some phosphorylated bands (~35 kDa for etk, 55 kDa for wzc, 40 kDa for yjjJ, and several bands between 50 and 70 kDa for nrdD). However, argG and dgoD did not show observable differences (Figure 3C). Total protein staining was done with CBB as a loading control as shown in Figure S2.

### Autophosphorylation of nrdD *In Vitro*

**Figure 4:**
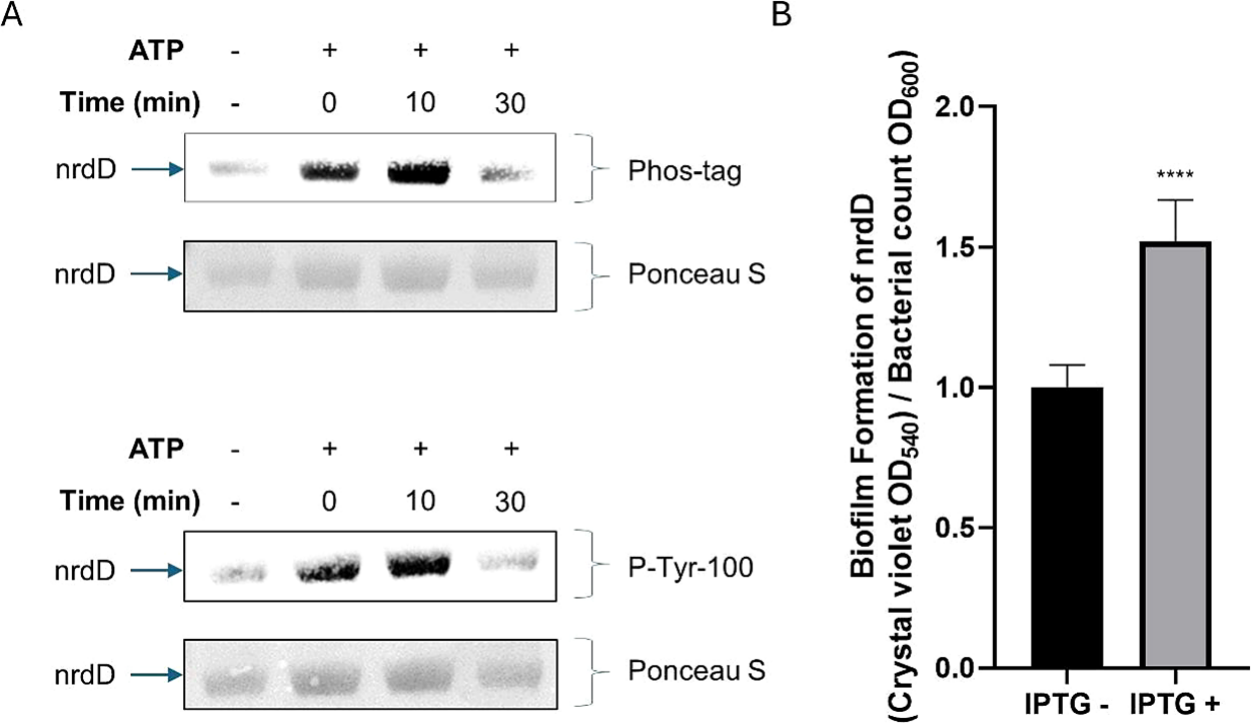
Contribution of nrdD to autophosphorylation and biofilm formation. (A) Purified nrdD was treated with a kinase buffer supplemented with ATP for various time points. The level of phosphorylation was quantified by Phos-tag and P-Tyr-100 antibody with the SDS–PAGE. The same membrane was stained for Ponceau S for loading controls. (B) Biofilms were quantified based on the crystal violet staining in nrdD clones with or without IPTG induction. Data were analyzed from five replicates using an unpaired two-tailed *t* test, ****p < 0.0001.

Among 4 candidates validated in cell lysates, nrdD previously known as anaerobic ribonucleoside-triphosphate reductase novel kinase showed phosphorylation activities. Since most BY-kinases are autokinases, we analyzed nrdD for the autophosphorylation activities (Figure 4A). The autophosphorylation was observed immediately after ATP addition (0 min), increased more after 10 min and decreased after 30 min. Both Phos-tag and P-Tyr-100 antibody staining showed similar phosphorylation patterns (Figure 4A). The total protein staining was done with Ponceau S as the loading control.

### nrdD Involved in Biofilm Formation

BY-kinases are usually involved in biofilm formation in *E. coli* that is important in drug resistance and bacterial survival under harsh environments.^25^ Therefore, we cultured nrdD clones with or without IPTG induction and performed biofilm assays by crystal violet staining. After 18 h of static growth, a significant increase in biofilm was observed in IPTG compared to that in non-IPTG (Figure 4B). The biofilm formation was normalized to the bacterial count = crystal violet at OD_540_/bacterial concentration at OD_600_. A similar biofilm assay was done in the WT and showed no differences in IPTG induction (data not shown).

### Sequence Similarities of nrdD

Since nrdD was important in biofilm formation, we analyzed the sequence similarity of amino acids in every species, especially in bacteria. Based on the BLASTP search, various bacteria and a T4 phage have proteins similar to nrdD. Most of the species were Gram (−) bacteria, except two Gram (+) bacteria and one T4 phage. The polyphyletic tree, percent identity, and *E* value for nrdD from each species are shown in Figure S4A. The Alphafold structure of nrdD proteins from *E. coli* K-12 and other bacterial homologs are listed in Figure S4B–G. UniProt protein IDs of the nrdD in different species were also listed in Table S4. The low *E* value of the nrdD proteins (E-threshold 1) indicated that nrdD was highly conserved.

### *E. coli* Proteome Microarray Revealed Substrates of nrdD

After we observed that nrdD was an autokinase and phosphorylated several lysate protein bands on Western blot, we planned to interrogate the substrates of nrdD by using an *E. coli* proteome microarray with thousands of purified *E. coli* proteins. By treating the *E. coli* proteome microarray with nrdD and performing the *in vitro* kinase assay with or without ATP, we may reveal the substrates for nrdD (Figure S5A). After two repeats and statistical analysis, we identified 33 hits with elevated phosphorylation levels in the presence of ATP (Table S5).

Based on the differences with or without ATP, the top-ranked substrate was ssuD on the *E. coli* proteome microarrays (Figure S5B,C). We also tested the *in vitro* kinase assay between nrdD and ssuD with ELISA format and displayed similar results (Figure S5D). Finally, we demonstrated ssuD phosphorylation by nrdD with Western blot analysis. The phosphorylation level was only elevated in ssuD treated with both ATP and nrdD (Figure S5E). The quantification of ssuD phosphorylation based on Pro-Q Dimond blot also showed significance in the presence of ATP and nrdD (Figure S5F).

## Discussion

In this study, we adapted the single gene overexpression library (ASKA) and established the world’s first *E. coli* lysate array (ECLA) for high-throughput screening of tyrosine phosphorylation events. Among 6 candidates identified by ECLA, etk and wzc were already known BY-kinases^2,3^ and identified again in this study. The successful identification of etk and wzc strongly supports the robustness and usefulness of the ECLA. nrdD is being reported here for the first time as a BY-kinase because it has autophosphorylation activity and transphosphorylates other substrates. We also tested phosphorylation of WT lysates by nrdD with phosphoserine/threonine and phospho-histidine antibodies. However, we observed phosphorylation with phosphotyrosine antibodies only (Figure S3). This also suggests that nrdD mainly exhibited tyrosine phosphorylation. yjjJ is a known serine/threonine kinase; however, it was still found on our candidate BY-kinase list. The reason might be due to its cross-phosphorylation activity on some proteins’ tyrosine residues, which was also reported before.^26^ dgoD seemed to induce some change as it increased the intensity of a phosphorylated band in lysates; however, in *E. coli* proteome microarray profiling, it did not show any of the potential substrates (data not shown). argG was also among our candidates, but we did not observe any phosphorylation activity. One explanation for that might be the nonspecific binding of the phosphotyrosine antibodies to these proteins. Another reason might be explained as these proteins might be highly phosphorylated substrates or an overexpressed protein triggering a downstream mechanism.

Proteome microarray is a useful tool for global analysis of protein phosphorylation *in vitro* to identify substrates of known kinases.^27^ We used the *E. coli* proteome microarray and found 33 substrates of nrdD by performing the *in vitro* kinase assay. ssuD was the highest-ranking substrate candidate, which is the alkanesulfonate monooxygenase enzyme in *E. coli*. The phosphorylation of ssuD by nrdD was verified with Western blot and solid capture assays. The substrate list of nrdD in Table S5 shows various oxidoreductases such as ssuD that were the targets of nrdD. This supports the function that the function of nrdD might also be related to a protective strategy against oxidative stress.

Our *in vivo* functional studies revealed that nrdD overexpression significantly increased biofilm production in *E. coli*. Biofilm formation is another importance for BY-kinases in *E. coli*, as they contribute to the biofilm and exopolysaccharide production.^25^ Cendra et al. have shown that Δ*nrdD* and Δ*nrdD* Δ*nrdE* knockout strains have decreased biofilm formation.^28^ They also showed that the level of expression of nrdD was elevated in biofilm-forming *E. coli*. Among the substrates of nrdD (Table S5), we found that exopolysaccharide-associated wcaC, fcl, and kpsE were among nrdD substrates.^29−31^ These results also support the idea that nrdD directly contributes to the formation of exopolysaccharides in *E. coli*.

The multiple protein sequence alignments made with nrdD in other species showed that nrdD is conserved among the bacteria. nrdD is a Class III anaerobic ribonucleoside-triphosphate reductase. It is known for catalyzing the conversion of ribonucleotides into deoxyribonucleotides, which are required for DNA synthesis and repair.^32^ Most of the known BY-kinases have the P-loop structure for ATP binding.^33^ Unlike these BY-kinases, nrdD has an ATP-cone structure for ATP binding. Structurally, ATP-cone seems to be conserved among nrdD in several bacterial homologs (Figure S4B–G). This domain was also found in kinases such as phosphoglycerate kinase 2 (PGK2).^34^ Therefore, our finding suggests that nrdD belongs to a new family of BY-kinases. Its phosphorylation mechanism should be investigated with nrdD from other species to determine whether the kinase activity is also conserved among species.

In conclusion, ECLA works as a practical approach to profile phosphorylation in cell lysates, as it reflects *in vivo* conditions to interrogate gene functions in high throughput. Our results show that ECLA might be a useful alternative to *in silico* surveys to expand the kinome of *E. coli*. One of the limitations of ECLAs might be the inhibition of culture growth due to the overexpressed proteins. If the growth and viability decrease, this might affect the detection of the PTMs in the lysates. However, this might be overcome by optimizing the IPTG induction of the cultures having slow growth. The other limitation is that the interrogation of PTMs on ECLA depends on the antibody quality. ECLA might be a useful tool to apply to different PTMs and discover various novel enzymes in *E. coli*. These studies are ongoing in our laboratory, and those PTM enzymes will be investigated in the future.

## Data Availability

The data sets generated in this study are available from the corresponding author upon reasonable request.

## Supporting Information Available

List of ECLA spots with elevated tyrosine phosphorylation ranking, phylogenetic tree, list and figures of nrdD protein from other bacteria, list of nrdD substrates obtained from *in vitro* kinase assay on *E. coli* proteome microarray, *in vitro* kinase assay results on *E. coli* proteome array to verify ssuD as a substrate, and additional supporting Western blot images (PDF)
