# Supplementary material for: Reverse-Phase Protein Microarrays for Overexpressed Escherichia coli Lysates Reveal a Novel Tyrosine Kinase
Source: Anal Chem. 2024 Apr 29;96(21):8721–9. doi: 10.1021/acs.analchem.4c00965 (PMC11140677; doi:10.1021/acs.analchem.4c00965)
Supplement: Supplementary file 1 — ac4c00965_si_001.pdf [file ac4c00965_si_001.pdf]

Supporting Information

Reverse Phase Protein Microarrays for Overexpressed *Escherichia Coli* Lysates Reveal a Novel Tyrosine Kinase

Batuhan Birol Keskin[1]#, Chien-Sheng Chen[2,3], Pei-Shan Tsai[1], Pin-Xian Du[1], John Harvey M. Santos[1,4], Guan-Da Syu[1,5,6]*

[1]Department of Biotechnology and Bioindustry Sciences, National Cheng Kung University, Tainan 701, Taiwan

[2]Department of Food Safety/Hygiene and Risk Management, College of Medicine, National Cheng Kung University, Tainan, Taiwan

[3]Institute of Basic Medical Sciences, College of Medicine, National Cheng Kung University, Tainan, Taiwan

[4]The University of Queensland, Centre for Animal Science, Queensland Alliance for Agriculture and Food Innovation, Brisbane, QLD, Australia

[5]International Center for Wound Repair and Regeneration, National Cheng Kung University, Tainan 701, Taiwan

[6]Medical Device Innovation Center, National Cheng Kung University, Tainan 701, Taiwan

#First Author

*Corresponding Author

Guan-Da Syu, PhD

Room 89A07, Department of Biotechnology and Bioindustry Sciences,

No.1, University Road, Tainan City 701, Taiwan

Telephone Number: +886-6-275-7575#58231

Fax Number: +886-6-276-6490

E-mail address: guanda@gs.ncku.edu.tw

Table of Contents

Table S1. List of ECLA spots with elevated tyrosine phosphorylation with P-Tyr-100 antibody **S3**

Table S2. List of ECLA spots with elevated tyrosine phosphorylation with PY20 anti-phosphotyrosine antibody **S4**

Table S3. List of overlapping ECLA spots obtained from two different anti-phosphotyrosine antibody assays **S5**

Table S4. Organisms used in nrdD protein sequence alignment **S6**

Table S5. List of nrdD substrates obtained from *in vitro* kinase assay on *E. coli* proteome microarray **S7**

Figure S1. Candidate hits ranked by phosphotyrosine antibody PY20 assays **S8**

Figure S2. Total protein staining for *in vitro* kinase assay of purified candidates added to WT lysates **S9**

Figure S3. *In vitro* kinase assays of nrdD and stained with different site-specific phosphorylation antibodies **S10**

Figure S4. Phylogenetic and structural similarities among bacterial nrdD proteins **S11**

Figure S5. *In vitro* kinase assay on *E. coli* proteome array to discover substrates of nrdD **S13**

Table S1. List of ECLA spots with elevated tyrosine phosphorylation with P-Tyr-100 antibody (1.5 SD cut-off).

Rank	Overexpressed Protein	pTyr100 F.I. $_{mean}$ + SD	WT_{mean} + SD	ΔF.I.	p-value
1	etk	76177.21	1368.11	74809.11	1.03E-40
2	folE	15057.13	1368.11	13689.02	2.76E-26
3	fdhD	8292.98	1368.11	6924.87	1.49E-31
4	wzc	7359.17	1368.11	5991.06	8.45E-32
5	rbsA	5613.74	1368.11	4245.63	1.07E-26
6	yjfI	5744.28	1368.11	4376.17	3.98E-25
7	dgoD	6083.77	1368.11	4715.66	3.61E-22
8	argG	5156.02	1368.11	3787.91	1.60E-24
9	ebgR	4384.91	1368.11	3016.80	4.79E-20
10	yjjJ	4247.75	1368.11	2879.65	1.01E-19
11	ybaB	4059.24	1368.11	2691.13	1.31E-19
12	iscS	4006.95	1368.11	2638.84	9.62E-19
13	speA	3774.68	1368.11	2406.57	1.71E-17
14	lysU	3942.47	1368.11	2574.36	3.05E-17
15	prmB	3961.52	1368.11	2593.41	2.79E-16
16	yjeB	3732.55	1368.11	2364.44	2.48E-16
17	purF	3811.44	1368.11	2443.33	6.68E-16
18	yjjK	3808.23	1368.11	2440.13	1.50E-15
19	nrdD	3457.39	1368.11	2089.28	1.26E-14
20	nusA	3559.08	1368.11	2190.97	6.32E-14
21	potA	3645.32	1368.11	2277.21	1.84E-13
22	yfbG	3939.17	1368.11	2571.07	3.07E-11
23	hypF	3188.06	1368.11	1819.95	6.92E-13
24	purL	3574.78	1368.11	2206.67	4.51E-12
25	carB	3755.85	1368.11	2387.74	1.63E-11
26	cbrC	3372.17	1368.11	2004.06	7.71E-12
27	glgP	3071.72	1368.11	1703.61	7.25E-12
28	nrdF	3294.40	1368.11	1926.29	1.75E-11
29	ydiU	3210.96	1368.11	1842.85	1.20E-11
30	livF	2960.51	1368.11	1592.40	2.88E-11
31	narY	3246.93	1368.11	1878.82	9.13E-11
32	ybaT	2919.10	1368.11	1550.99	9.62E-11

Table S2. List of ECLA spots with elevated tyrosine phosphorylation with PY20 anti-phosphotyrosine antibody (1.5 SD cut-off)

Rank	Overexpressed Protein	PY20 F.I. $_{mean}$ + SD	WT$_{mean}$ + SD	∆F.I.	p-value
1	etk	100049.20	1286.79	98762.40	5.94E-24
2	dgoD	13239.48	1286.79	11952.69	1.08E-26
3	wzc	7704.57	1286.79	6417.78	5.64E-31
4	yjjJ	6604.89	1286.79	5318.10	2.44E-14
5	argG	3282.17	1286.79	1995.38	3.25E-14
6	ygbM	2996.95	1286.79	1710.16	2.83E-13
7	nrdD	2815.85	1286.79	1529.06	1.14E-11

Table S3. List of overlapping ECLA spots obtained from two different anti-phosphotyrosine antibody assays.

No	Overlapped Hits	UniProt ID	Antibody #1 Rank	Antibody #2 Rank
1	etk	P38134	1	1
2	wzc	P76387	4	3
3	dgoD	Q6BF17	7	2
4	argG	P0A6E4	8	5
5	yjjJ	P39410	10	4
6	nrdD	P28903	19	6

Table S4. Organisms used in nrdD protein sequence alignment.

Number	Organism	Uniprot ID
1	*Escherichia coli* (strain K12)	P28903
2	*Shigella flexneri*	*A0A0H2W0U1*
3	*Escherichia coli* O157:H7	Q8XCE2
4	*Escherichia coli* O45:K1 (strain S88)	B7MLP6
5	*Salmonella typhimurium* (strain LT2)	Q9L646
6	*Enterobacteriaceae bacterium* (strain FGI 57)	L0M9D7
7	*Enterobacter lignolyticus* (strain SCF1)	E3GCI4
8	*Klebsiella michiganensis*	A0A7H9H0R8
9	*Shewanella oneidensis* (strain MR-1)	Q8EDC8
10	*Erwinia piriflorinigrans* CFBP 5888	V5Z386
11	*Clostridia bacterium*	A0A6N7M883
12	*Haemophilus influenzae* (strain ATCC 51907)	P43752
13	*Fontibacillus phaseoli*	A0A369BDU2
14	*Paenibacillus lutimineralis*	A0A3Q9IFY9
15	*Gardnerella vaginalis* (strain ATCC 14019)	E3D8W3
16	*Chitinibacter fontanus*	*A0A7D5V8W0*
17	Enterobacteria phage T4	P07071

Table S5. List of nrdD substrates obtained from *in vitro* kinase assay on *E. coli* proteome microarray

Rank	Protein Name	UniProt ID	Average ± SD $_{(ATP +)}$	Average + SD $_{(ATP -)}$	(ATP+) – (ATP-)	p-value
1	ssuD	P80645	12595.75 ± 65.41	10009.00 ± 228.40	2586.75	0.0275
2	folM	P0AFS3	2144.50 ± 305.47	824.50 ± 195.87	1320.00	0.0494
3	wcaC	P71237	2544.75 ± 215.31	1406.50 ± 200.82	1138.25	0.0322
4	hdeA	P0AES9	1039.25 ± 63.99	232.5 0± 63.64	806.75	0.0062
5	fcl	P32055	1065.25 ± 141.77	293.75 ± 107.83	771.50	0.0302
6	gadB	P69910	1628.50 ± 103.94	1044.00 ± 45.96	584.50	0.0460
7	yicG	P0AGM2	1772.25 ± 70.36	1193.00 ± 14.85	579.25	0.0458
8	eutI	P77218	837.25 ± 76.72	293.00 ± 86.97	544.25	0.0228
9	ypjA	P52143	606.00 ± 52.33	108.50 ± 67.18	497.50	0.0170
10	ydaL	P76053	687.75 ± 3.18	193.25 ± 49.85	494.50	0.0445
11	nuoC	P33599	1072.25 ± 8.84	579.25 ± 42.78	493.00	0.0320
12	ydhF	P76187	647.25 ± 99.35	174.50 ± 69.30	472.75	0.0399
13	fbp	P0A993	1166.50 ± 6.36	695.75 ± 36.42	470.75	0.0300
14	citX	P0A6G5	673.50 ± 41.01	214.50 ± 4.95	459.00	0.0376
15	dksA	P0ABS1	622.75 ± 5.30	205.00 ± 43.13	417.75	0.0435
16	ycjU	P77366	610.25 ± 61.16	222.25 ± 49.14	388.00	0.0223
17	rffC	P27832	410.50 ± 40.31	27.25 ± 8.13	383.25	0.0398
18	rcsB	P0DMC7	678.50 ± 69.30	299.50 ± 43.13	379.00	0.0345
19	cheW	P0A964	485.25 ± 68.94	113.50 ± 61.52	371.75	0.0304
20	ydaE	P38394	406.50 ± 22.63	39.75 ± 40.66	366.75	0.0177
21	asnC	P0ACI6	447.75 ± 33.59	89.25 ± 3.18	358.50	0.0404
22	ydhS	P77148	624.00 ± 79.20	293.50 ± 59.40	330.50	0.0486
23	yncG	P76117	461.50 ± 20.51	135.25 ± 12.37	326.25	0.0063
24	rpsB	P0A7V0	490.75 ± 36.42	170.75 ± 40.66	320.00	0.0148
25	hscC	P77319	483.25 ± 41.37	179.25 ± 51.97	304.00	0.0260
26	yfiF	P0AGJ5	399.50 ± 46.67	111.75 ± 60.46	287.75	0.0382
27	amn	P0AE12	389.75 ± 30.05	109.25 ± 43.49	280.50	0.0237
28	yaiX	P75697	397.75 ± 0.35	118.00 ± 20.51	279.75	0.0329
29	entB	P0ADI4	476.25 ± 42.78	200.75 ± 39.95	275.50	0.0221
30	dcd	P28248	381.50 ± 24.75	112.00 ± 47.38	269.50	0.0385
31	nrfA	P0ABK9	376.75 ± 39.95	118.50 ± 47.38	258.25	0.0295
32	dnaG	P0ABS5	319.25 ± 2.47	63.50 ± 4.95	255.75	0.0016
33	kpsE	P62586	348.25 ± 55.51	95.00 ± 54.45	253.25	0.0441

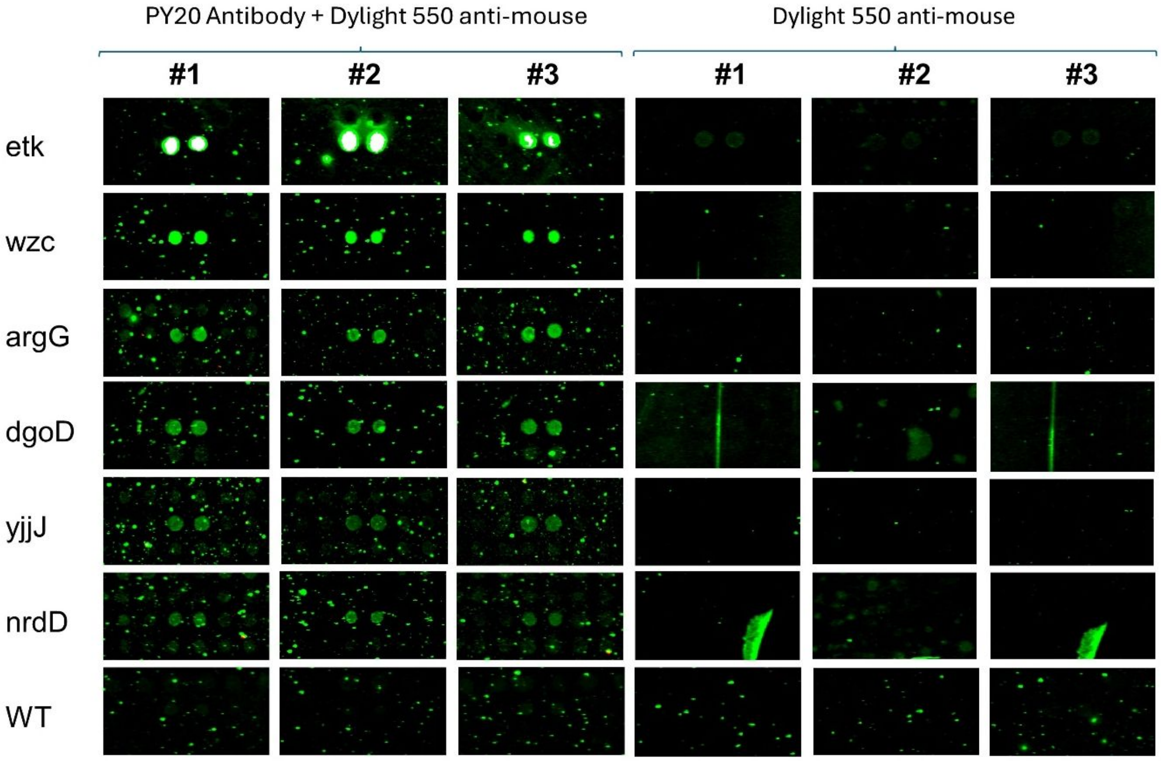

Figure S1. Candidate hits ranked by phosphotyrosine antibody PY20 assays.

The images from the three assays were done with PY20 antibody followed by fluorescent-labeled anti-mouse and three assays were done with fluorescent-labeled anti-mouse as blanks. The WT lysate spots were listed as baseline phosphorylation.

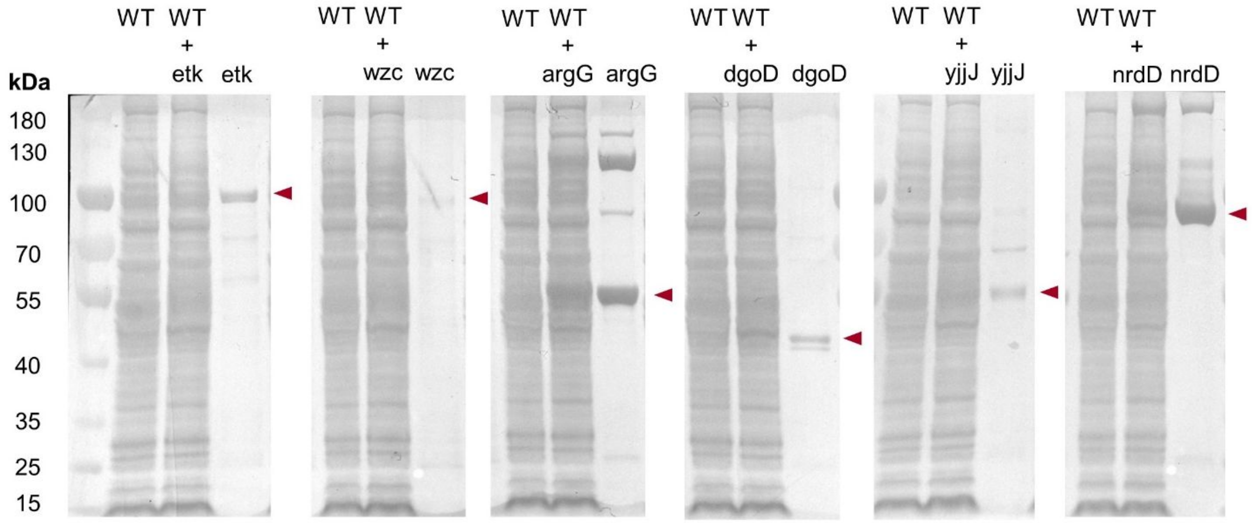

Figure S2. Total protein staining for *in vitro* kinase assay of purified candidates added to WT lysates.

Each purified candidate was incubated with or without WT lysates in the kinase buffer supplemented with ATP before analyzing by SDS-PAGE. Coomassie Brilliant Blue staining was performed to show purified proteins and the total proteins in WT lysates.

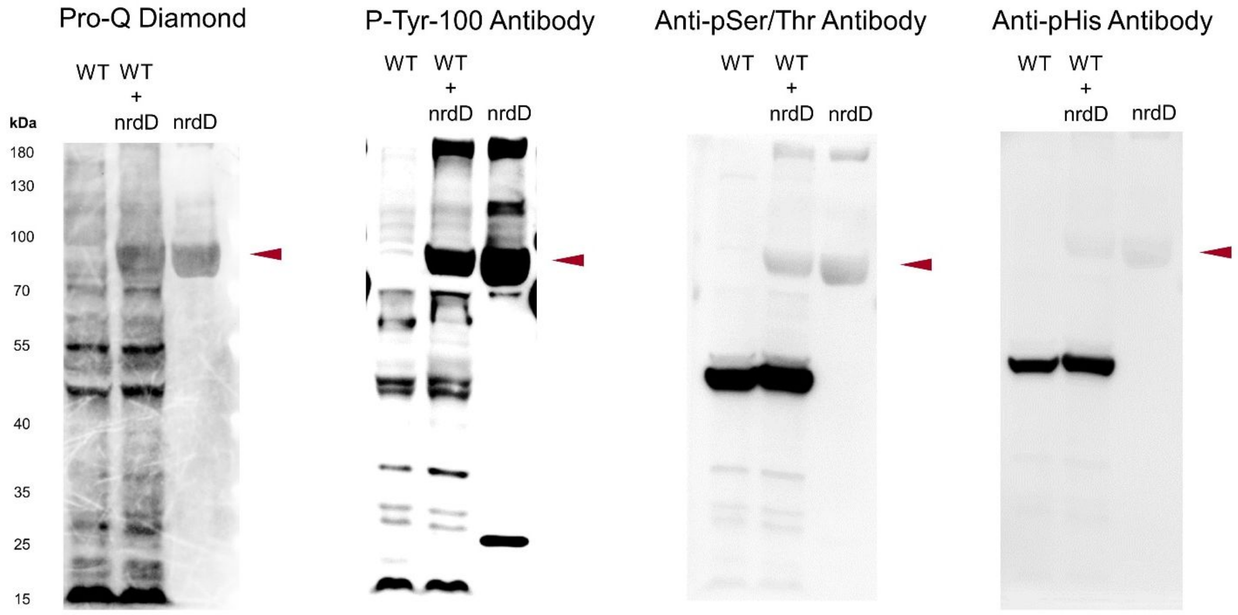

Figure S3. *In vitro* kinase assays of nrdD and stained with different site-specific phosphorylation antibodies.

WT lysate was incubated with or without nrdD in the kinase buffer supplemented with ATP, ran the SDS-PAGE, and analyzed by Pro-Q Diamond. Tyrosine, serine/threonine, and histidine site-specific phosphorylation blots were compared. Red arrows indicated the purified nrdD proteins.

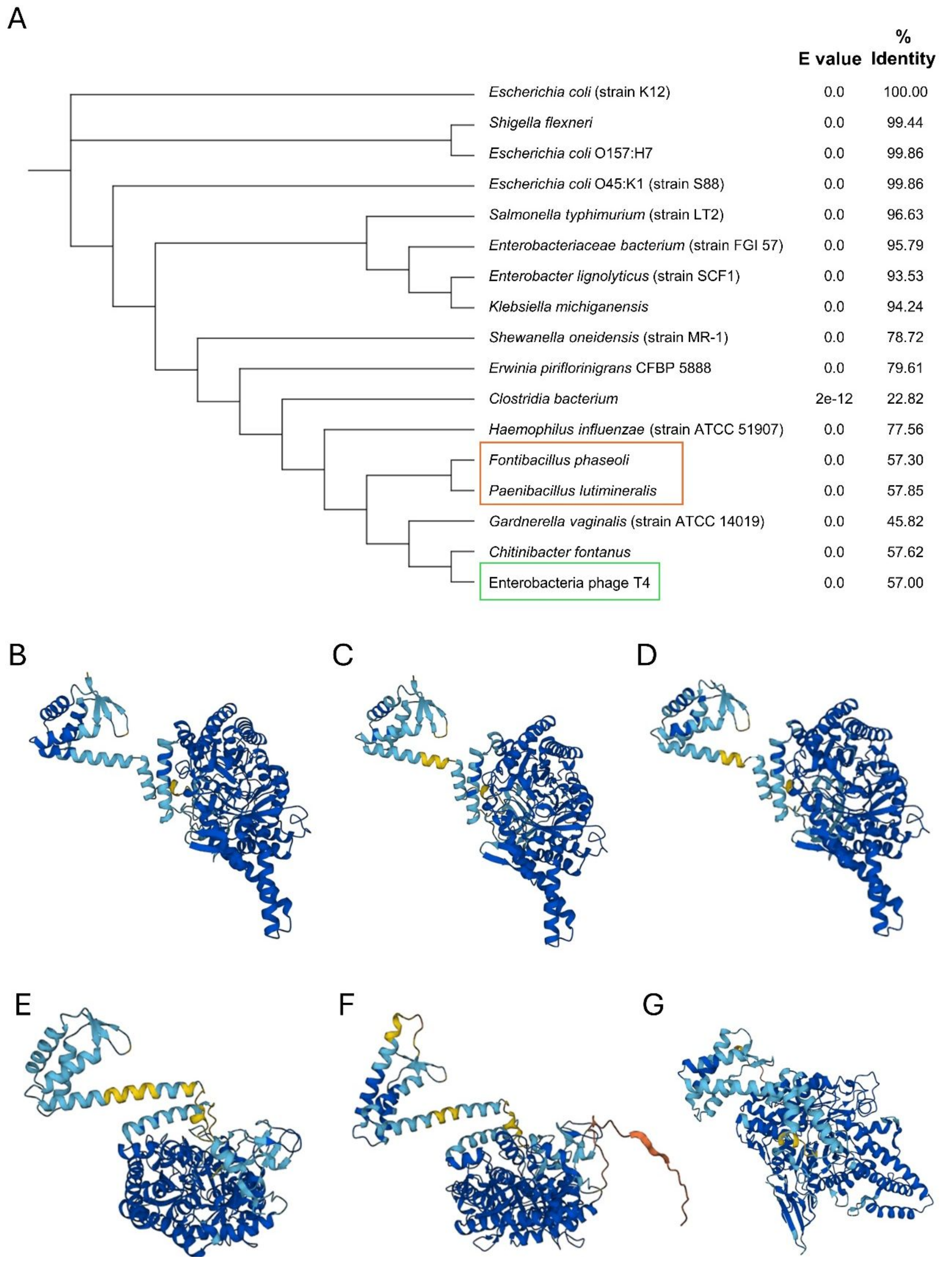

Figure S4. Phylogenetic and structural similarities among bacterial nrdD proteins

(A) The alignments of nrdD protein sequences from *E. coli* and other organisms. The phylogenetic tree was constructed by IQ-TREE web server and visualized by i-TOL. The percent identity and E value of each organism's nrdD were evaluated by NCBI BlastP Suite. Gram positives were indicated in the orange box and T4 bacteriophage in the green box. Alphafold structures of the nrdD proteins of (B) *Escherichia coli* (strain K12) (C) *Klebsiella michiganensis* (D) *Shewanella oneidensis* (strain MR-1) (E) *Fontibacillus phaseoli* (F) *Chitinibacter fontanus* (G) *Gardnerella vaginalis* (strain ATCC 14019) are shown. ATP cone structures can be observed on the upper left part of the proteins.

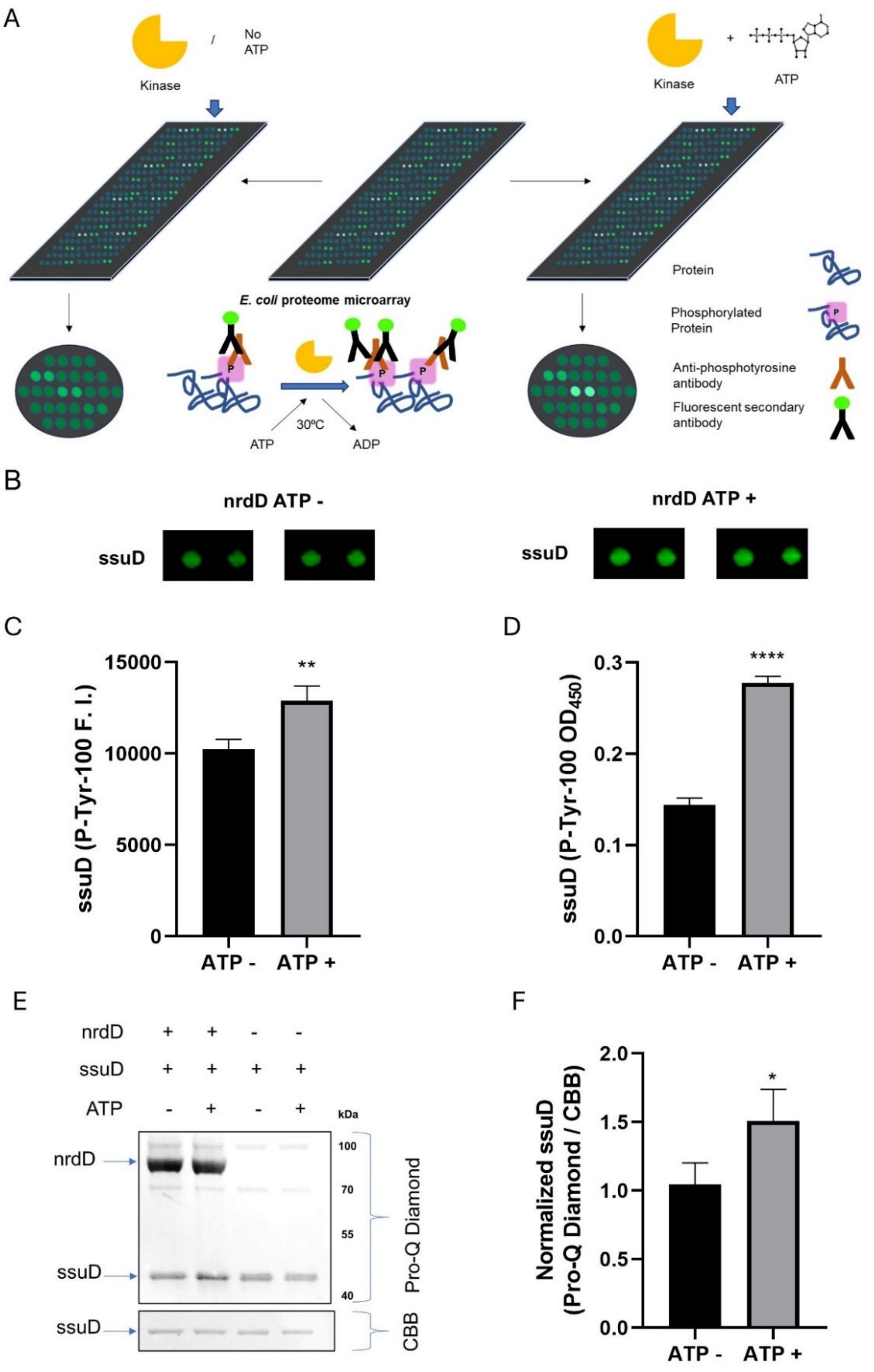

Figure S5. *In vitro* kinase assay on *E. coli* proteome array to discover substrates of nrdD

The comparison of nrdD with or without ATP was used to quantify the kinase activity. (A) *E. coli* proteome microarrays were incubated with purified nrdD with or without ATP and visualized with anti-pTyr-100 staining. (B) The images from duplicate microarray assays of the top-ranked substrates with or without ATP addition. (C) The quantification of the fluorescence intensities on the microarrays. (D) *In vitro* kinase assay on the microplates by immobilizing ssuD, incubated with nrdD with or without ATP, stained with anti-pTyr-100, and quantified by anti-mouse HRP. Experiments were done in triplicates. (E) Western blot image of kinase reaction of nrdD and ssuD with or without ATP using Pro-Q Diamond staining. Coomassie Brilliant Blue (CBB) was used to show the total protein. (F) Triplicate blotting results were quantified based on the ratio between Pro-Q Diamond and CBB band intensities. Data were analyzed with an unpaired two-tailed t-test * $p < 0.05$, ** $p < 0.01$, and **** $p < 0.0001$ (ATP - versus ATP +).
